# Predicting the Need for Intensive Care Unit Treatment After Successful Transcatheter Edge-to-Edge Mitral Valve Repair

**DOI:** 10.3390/jcm14072167

**Published:** 2025-03-22

**Authors:** Felix Ausbuettel, Dieter Fischer, Fares Kano, Nikolaos Patsalis, Christin Fichera, Dimitar Divchev, Carlo-Federico Fichera

**Affiliations:** 1 Department of Cardiology, University Hospital Marburg, Baldingerstraße, 35043 Marburg, Germany; 2 Medical Clinic II, Department of Cardiology, Hospital Rheine, Frankenburgstraße, 48431 Rheine, Germany; 3 Faculty of Medicine, Justus-Liebig University Giessen, Ludwigstraße 23, 35390 Giessen, Germany; 4 Clinic and Polyclinic for Internal Medicine B, University Hospital Greifswald, Ferdinand-Sauerbruch-Straße, 17475 Greifswald, Germany; 5 Department of Cardiology, County Hospital Loerrach, Spitalstraße 25, 79539 Loerrach, Germany

**Keywords:** mitral valve repair, MitraClip, PASCAL, intensive care unit, heart failure, mitral regurgitation

## Abstract

**Background/Objectives:** Transcatheter edge-to-edge mitral valve repair (M-TEER) has emerged as an efficacious treatment modality among patients at high perioperative risk. Given the steady increase in procedures and the limited capacity for intensive care, there is a need to identify patients at high risk for postinterventional intensive care. **Methods:** All patients who underwent M-TEER between 2014 and 2023 were investigated. The intensive care unit (ICU) stay ended when patients met all the following criteria: no further need for catecholamine support, no oxygen requirement > 6 L O2/min, no indication for renal replacement therapy, and no delirium or relevant bleeding. Uni- and multivariable logistic regression analyses were used to identify independent predictors of the need for ICU treatment. **Results:** In total, 33% of patients (62/183) had an indication for ICU treatment after M-TEER. Patients with an indication for ICU treatment had significantly lower survival rates three years after M-TEER (37.4% [23/62] vs. 61.6% [75/121], *p* < 0.001) than patients without an ICU indication. A EuroSCORE II of >10% (OR 2.6, 95% CI 1.3–5.4, *p* = 0.006), a MitraScore of >3 (OR 2.5, 95% CI 1.2–5.2, *p* = 0.02), and a hospital stay of >5 days before M-TEER (OR 3.2, 95% CI 1.6–6.4, *p* < 0.001) were independently associated with the need for ICU treatment. **Conclusions:** One-third of the patients were indicated for ICU treatment, which was associated with a high mortality rate. On the basis of these predictors of required ICU care, tailored treatment strategies can be developed to improve treatment outcomes.

## 1. Introduction

Transcatheter edge-to-edge mitral valve repair (M-TEER) constitutes a minimally invasive and efficacious treatment modality for patients suffering from high-grade mitral regurgitation despite optimized heart failure therapy that are rejected for surgery due to increased perioperative risk [1,2,3,4,5,6].

Previous studies have shown that the majority of M-TEER patients are transferred to the intensive care unit (ICU) postintervention [7,8]. These patients are treated mainly under general anesthesia and require further treatment with inotropics, vasopressors, or mechanical ventilation [9,10]. Owing to the difficulty in predicting postinterventional care, most patients are transferred to an ICU after successful M-TEER [11]. Even though analgosedation is gaining in popularity for M-TEER, a proportion of patients still require increased awareness and ICU treatment following the M-TEER intervention. However, further data on the requirement for ICU treatment following M-TEER are lacking to date. These data concern both the proportion of patients requiring ICU treatment and the distribution of ICU indications. With respect to the constantly increasing number of interventions combined with the limited ICU capacity, there is a need to identify patients who need postinterventional ICU therapy [12]. The aim of this study was to shed light on the proportion of patients requiring ICU treatment and their respective indications. We also sought to investigate independent factors that correlate with the need for ICU treatment after M-TEER intervention.

## 2. Materials and Methods

In this monocentric observational cohort study, all patients who underwent M-TEER between May 2014 and May 2023 were investigated. All patients were not considered for surgical mitral valve reconstruction at the interdisciplinary heart team conference, which consisted of cardiac surgeons and interventional cardiologists. Anatomical suitability and the exclusion of any contraindications for M-TEER were previously carried out using transesophageal echocardiography (TOE). The M-TEER procedure was primarily performed under analgosedation and TOE guidance using either a MitraClip^®^ (Abbott Vascular, Chicago, IL, USA) or a PASCAL^TM^ system (Edwards Lifesciences, Irvine, CA, USA). The respective implantation techniques have already been described [5,13,14]. Patients who were converted from analgosedation to general anesthesia during the procedure according to the decision of the interventional team were also included in the statistical analysis. Analgosedation was performed with morphine, midazolam, and propofol, and in cases of general anesthesia, the standardized protocol included sufentanil. Procedural complications were recorded in a standardized manner using the Mitral Valve Academic Research Consortium (MVARC) classification [15].

Monitoring during intervention included invasive arterial blood pressure measurement, 4-lead electrocardiography, and monitoring of peripheral O2 saturation. At the end of the procedures, the patients were transferred routinely to the ICU. Postinterventionally, the patients received dual antiplatelet therapy or anticoagulation with vitamin K antagonists or factor Xa inhibitors in combination with a P2Y12 receptor inhibitor on the basis of the indication for oral anticoagulation owing to comorbidities. The ICU stay ended when the patient met all the following criteria: absence of catecholamines, oxygen demand < 6 L O2/min, no indication for dialysis, and no delirium or relevant bleeding according to the MVARC classification.

The primary endpoint of the study was the necessity for ICU treatment; the secondary endpoint was long-term mortality after M-TEER intervention. Accordingly, the patients were divided into two groups for the following statistical analysis: patients who did not actually require ICU treatment versus those who matched at least one criterion listed above and therefore had an indication for further ICU treatment.

### Statistical Analysis

Categorical variables are presented as absolute and relative frequencies; continuous variables are presented as the means and standard deviations for normally distributed variables and as medians and interquartile ranges for nonnormally distributed variables. The assumption of a normal distribution was verified using the Shapiro-Wilk test. Differences between the compared groups were evaluated in the case of categorical variables by when the expected cell size was <20 or the chi-square test when the expected cell size was ≥20. For continuous variables, Student’s *t* test was used to assess differences in normally distributed variables, and the Wilcoxon test was used for nonnormally distributed variables. The analysis of long-term survival was performed via the Kaplan-Meier method, and differences between groups were compared by using the log-rank test. Independent predictors of ICU treatment were identified via univariate logistic regression, as were independent predictors of mortality via univariate Cox regression analyses. The primary and secondary endpoints of the study were respectively defined as dependent variables in the logistic regression and Cox regression analysis. The other variables recorded as part of the study were tested for statistical significance as independent variables. All variables with a significant *p*-value were then included stepwise in the multivariable model. A two-sided *p* value of ≤0.05 was considered statistically significant. The statistical analyses were performed with R Studio V4.3.1 (R Foundation for Statistical Computing Platform, Vienna, Austria), including the packages “survival”, “survminer”, and “dplyr”, and GraphPad Prism 6.0 (GraphPad Software, La Jolla, CA, USA). The graphics were designed using R Studio V4.3.1 and BioRender.com (Science Suite Inc., Toronto, ON, Canada).

## 3. Results

### 3.1. Clinical Cohort Characteristics

A total of 183 patients were considered for further analysis during the observational period, 33% of whom (62/183) revealed an indication for further ICU treatment.

Patients with indications for further ICU treatment presented with more advanced stages of congestive heart failure with higher New York Heart Association (NYHA) stages (53.2% (33/62) vs. 31.4% (38/121), *p* = 0.006), higher NTproBNP levels (4158 ± 6502 pg/mL vs. 2531 ± 4532 pg/mL, *p* = 0.02), and higher rates of high-dose diuretic therapy, defined as ≥80 mg furosemide/d (56.5% (35/62) vs. 38.8% (47/121), *p* < 0.001). Consequently, patients with indications for further ICU treatment had higher values of the EuroSCORE II (15.3 ± 25.2% vs. 6.4 ± 7%, *p* < 0.001), STS risk score (9.2 ± 17.1% vs. 5.6 ± 5%, *p* < 0.001), and MitraScore (4.2 ± 1.2 vs. 3.2 ± 1.4, *p* < 0.001) than patients without indications for further ICU treatment.

With respect to the procedural data, patients who required further ICU therapy had significantly longer procedure times than those without ICU indications (107 ± 42 min vs. 90 ± 34 min, *p* = 0.009). There was also a higher rate of major adverse cardiovascular and cerebrovascular events (MACCE) among patients with indications for further ICU therapy than among patients without indications for ICU therapy (37.1% (23/62) vs. 0% (0), *p* < 0.001).

No significant differences were observed in the obtained echocardiographic parameters between the groups, whereas the invasive pulmonary artery systolic pressure (PASP) was significantly greater among patients with indications for further ICU treatment (72 ± 18 mmHg vs. 55 ± 16 mmHg, *p* < 0.001). The clinical, echocardiographic, and procedural cohort characteristics are presented in Table 1.

### 3.2. Indications for Further ICU Treatment

**Figure 1:**
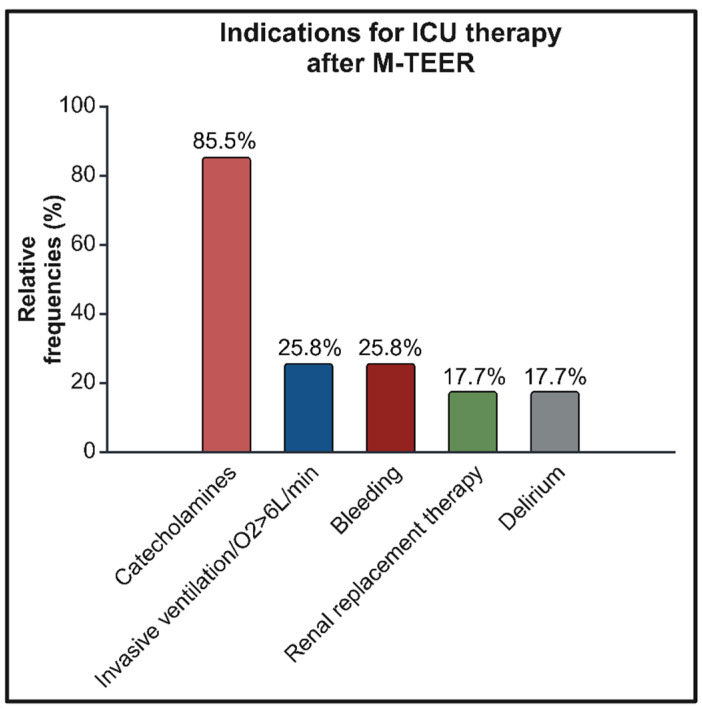
Overview of indications leading to ICU therapy after M-TEER. ICU—intensive care unit. M-TEER—transcatheter edge-to-edge mitral valve repair.

The predominant indication for further ICU therapy was postinterventional vasopressor or inotropic therapy in 85.5% (53/62) of patients. A total of 25.8% (16/62) of the remaining patients had respiratory insufficiency or relevant bleeding requiring either intervention or transfusion after M-TEER. Renal replacement therapy was needed in 17.7% (11/62) of patients, and an additional 17.7% (11/62) could not be discharged from the ICU due to delirium or comparative cognitive disorders. The distribution of indications for ICU therapy is shown in Figure 1.

After uni- and multivariable logistic regression analyses, a EuroSCORE II of >10% (OR 2.6, 95% CI 1.3–5.4, *p* = 0.006), a MitraScore of >3 (OR 2.5, 95% CI 1.2–5.2, *p* = 0.02), and a hospital stay of >5 days before M-TEER (OR 3.2, 95% CI 1.6–6.4, *p* < 0.001) were found to be predictive of the requirement for postinterventional ICU treatment after M-TEER intervention. The independent factors that correlate with a required ICU treatment after univariable logistic regression analysis are presented in Supplementary Table S1, and the factors after multivariable analysis are presented in Table 2.

### 3.3. Long-Term Outcomes

**Figure 2:**
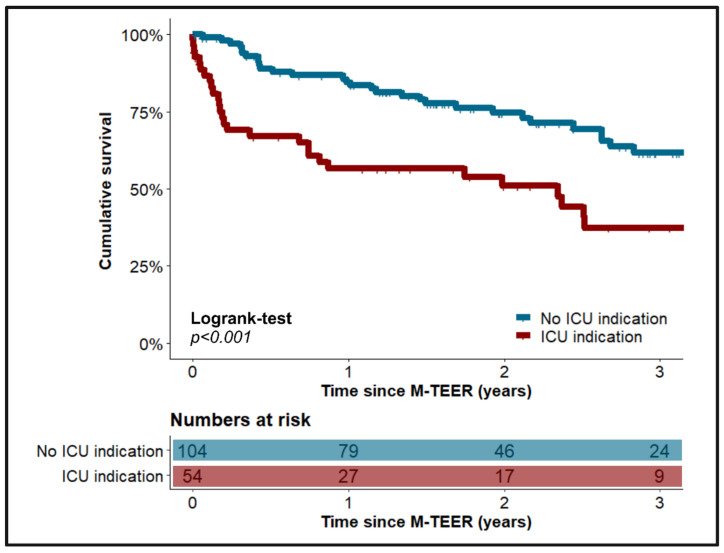
Comparison of long-term survival between patients with vs. without indication for further ICU therapy after successful M-TEER. ICU—intensive care unit. M-TEER—transcatheter edge-to-edge mitral valve repair.

Patients who required postinterventional ICU treatment exhibited significantly poorer survival at three years after M-TEER than patients without indications for further ICU therapy (37.4% (23/62) vs. 61.6% (75/121), *p* < 0.001), which is further presented in Figure 2.

The requirement for postinterventional ICU therapy emerged as an independent factor that correlated with mortality in the univariable Cox regression analysis (HR 2.3, 95% CI 1.4–3.7; *p* = 0.001). However, in the multivariable model, high-grade tricuspid regurgitation (TR; HR 4.2, 95% CI 2.1–8.2, *p* < 0.001), previous implantation of an implantable cardioverter-defibrillator (ICD; HR 2.2, 95% CI 1.4–3.7, *p* = 0.002), and a TAPSE/PASP ratio of >0.5 (HR 0.3, 95% CI 0.1–0.7, *p* = 0.004) were the predominant factors that correlated with mortality. The results of the univariable Cox regression analysis are given in Supplementary Table S2, and those of the multivariable Cox regression analysis are given in Table 3.

## 4. Discussion

The number of patients receiving M-TEER and the standardization of the procedure have increased over the years, but postinterventional care management usually remains center-dependent, generally including transfer of the patient to an ICU [16].

In this analysis, approximately two-thirds of the patients had no indication for ICU treatment. If there was an indication, it was primarily due to the catecholamine requirement. Only a small proportion of patients showed respiratory or renal insufficiency with indications for renal replacement therapy, as well as relevant bleeding or delirium. The need for pharmacological circulatory support was significantly lower in the present cohort than in previously reported data [11]. This might be related to the very low number of patients who received general anesthesia during the procedure. This kind of sedation method has been recommended in previous publications and continues to be performed as a standard procedure at various clinics, as it facilitates TOE guidance [17,18]. The performance of M-TEER under analgosedation has already been described as feasible [19,20], and the advantage of this approach lies in the rapid weaning from oxygen and catecholamines without the need for specific support by an anesthesiologist, enabling possible “fast-track” treatment after M-TEER.

In general, patients with postinterventional requirements for ICU therapy presented with greater cardiac morbidity than patients without ICU indications. Consequently, the mortality rate three years after M-TEER was significantly higher among patients with indications for further ICU therapy. In the context of the present study, factors that correlated with postinterventional ICU therapy were identified for the first time. The analysis was not adjusted using propensity score matching due to the limited number of patients. However, a bias in the results was considered unlikely, as there was no significant difference in the multivariate-identified mortality predictors between the compared cohorts.

The EuroSCORE II, STS risk score, and MitraScore have been established as perioperative risk scores for estimating postoperative mortality during interdisciplinary heart conferences [21,22,23]. The results of the present study likewise indicate a prediction of a required postinterventional ICU treatment following M-TEER. With regard to an increased MitraScore, a study by Rottländer et al. of the MITRA-PRO registry demonstrated a significantly increased mortality predominantly in patients with atrial functional MR, whereas residual MR was found to be a predictor of mortality in patients with ventricular functional MR. In the present study, persistent high-grade MR after M-TEER was found to be an independent factor that correlated with ICU care in the univariable logistic regression analysis, which, however, failed to reach the significance level in the multivariable analysis. As no distinction was made between patients with atrial and ventricular functional MR, no further conclusions can be drawn regarding the differing outcomes. Nevertheless, this study provides further confirmation that a low MitraScore and the minimum possible residual MR are associated with a favorable prognosis.

Regarding clinical factors that correlated with a need for ICU treatment, NYHA class IV, anemia with a hemoglobin (Hb) value of <10 g/dL, and chronic kidney failure, with a calculated glomerular filtration rate (GFR) of <45 mL/min, were identified. Resting dyspnea according to the NYHA classification has already been proven to be a surrogate parameter of worsening heart failure and an independent predictor of long-term mortality in M-TEER collectives and comparable collectives with congestive heart failure [24,25]. Anemia is known to worsen mortality, particularly in the case of iron deficiency and congestive heart failure with reduced LV function (HFrEF); simultaneously, postinterventional catecholamine requirements and bleeding requiring intervention can be accelerated, subsequently requiring ICU treatment [26]. The combination of chronic kidney failure and heart failure is known to cause and aggravate each one, subsequently limiting the long-term prognosis [27]. Additionally, an increased rate of incident decompensated heart failure was found among patients with GFR < 45 mL/min, which therefore complicated the course of treatment [28].

In terms of hemodynamic parameters, a pulmonary capillary wedge pressure (PCWP) of >30 mmHg and concomitant pulmonary arterial hypertension with a pulmonary vascular resistance (PVR) of >4 Wood units proved to be further factors that correlated with the need for ICU treatment. The former predictor, together with NYHA class IV, emphasizes the importance of adequate cardiac recompensation before M-TEER to prevent the need for ICU treatment. The latter illustrates the already known complicating influence of pulmonary hypertension on the morbidity of patients so that an increased probability of the need for ICU care has to be expected [29].

Finally, the number of days of in-hospital stay before intervention was associated with increased risk if patients were hospitalized for more than 5 days before M-TEER. This is probably because some of the patients analyzed were admitted electively and were hospitalized the day before the procedure. It can be assumed that elective patients presented with lower cardiac morbidity at the time of M-TEER, meaning that prolonged preprocedural hospitalization reflects a higher grade of deteriorated heart failure. In general, an elective procedure was associated with a better outcome than an urgent indication [30,31]. In the case of insufficient procedural success with residual high-grade mitral valve regurgitation, there was also an increased probability of requiring ICU treatment. Similar data have not been published to date, but this may also constitute a surrogate parameter of a complicated course with worsening heart failure.

In the multivariable logistic regression model from all the significant variables of the univariable analysis, the EuroSCORE II, MitraScore, and prolonged inpatient stay continued to emerge as significant factors associated with a required ICU treatment. Owing to the limited number of patients, it was not possible to add further variables, which should be investigated in larger and multicenter collectives in the future.

The need for ICU treatment following M-TEER was also a factor associated with long-term mortality in the univariable Cox regression analysis, which was consistent with the significant differences in long-term survival compared with patients who did not require ICU treatment. In the multivariate analysis, however, no corresponding correlation could be established for the reasons mentioned above.

## 5. Limitations

Several limitations must be considered when interpreting the results of the present study. Owing to the nature of an observational cohort study, no statements regarding any causalities can be drawn. Furthermore, no specific protocol for the postinterventional course was defined. The transferability of the present results to other multicenter collectives could be hindered due to the single-center nature of this study. In addition, treatment outcomes might have been influenced over the years due to gained expertise and therefore altered the statistical analysis. Regarding the identified predictors, a bias in the results due to uncollected and therefore unknown predictors cannot be ruled out. With regard to future studies, we therefore recommend conducting prospective multicenter studies on larger patient cohorts of current clinical care so that larger multivariable models can be formed. Depending on the heterogeneity of the cohorts, propensity score matching could also be used for adjustment of clinical characteristics. Nevertheless, highly relevant clinical endpoints have been developed to improve the outcomes of today’s “real-world” M-TEER patients.

## 6. Conclusions

The number of M-TEER interventions is constantly increasing and requires optimized postinterventional standards with better resource planning. ICU capacity is limited and must be utilized sensibly. One-third of the analyzed patients had an indication for ICU treatment, which was associated with a poorer long-term survival rate. Increased attention should therefore be paid to this third in order to improve the long-term survival. Furthermore, several risk factors for postinterventional intensive care treatment have been identified. Based on a EuroSCORE II of > 10%, MitraScore of > 3, and hospital stay of >5 days before M-TEER as independent factors associated with necessary ICU treatment, patients at risk can already be identified pre-interventionally. This approach may contribute to an improvement in the resource management and, moreover, in the overall prognosis of patients after M-TEER.

## Figures and Tables

**Table 1:** Clinical and procedural characteristics between patients with vs. without indication for ICU treatment after successful M-TEER.

Variable	Overall Cohort (*n* = 183)	No Indication for ICU (*n* = 121)	Indication for ICU (*n* = 62)	* p*-Value
Clinical cohort characteristics				
Age (years)	79 ± 7	79 ± 6	79 ± 7	0.9
Male sex	57.4% (105)	56.2% (68)	59.7% (37)	0.8
BMI (kg/m^2^)	27 ± 5	27 ± 5	27 ± 4	0.6
EuroSCORE II (%) *	7.5 ± 12	6.4 ± 7	15.3 ± 25.2	**<0.001**
STS risk score (%) *	6.4 ± 6.3	5.6 ± 5	9.2 ± 17.1	**<0.001**
MitraScore	3.6 ± 1.4	3.2 ± 1.4	4.2 ± 1.2	**<0.001**
COPD	24% (44)	22.3% (27)	27.4% (17)	0.5
CAD	73.8% (135)	71.1% (86)	79% (49)	0.3
Pacemaker *Prior CRT* *+ ICD*	29.5% (54) *10.4% (19)* *20.2% (37)*	28.9% (35) *13.2% (16)* *20.6% (25)*	30.6% (19) *20.9% (13)* *19.4% (12)*	0.9 *0.3* *0.08*
Diabetes mellitus	33.9% (62)	33.1% (40)	35.5% (22)	0.7
Arterial hypertension	88.5% (162)	87.6% (106)	90.3% (56)	0.8
Prior CAB-OP	21.9% (40)	19.8% (24)	25.8% (16)	0.4
Prior PCI	47% (86)	52.9% (64)	67.7% (42)	0.06
Previous stroke	11.5% (21)	13.2% (16)	8.1% (5)	0.3
Atrial fibrillation	72.1% (132)	71.1% (86)	74.2% (46)	0.7
PAD	13.7% (25)	10.7% (13)	19.4% (12)	0.1
NYHA III NYHA IV	56.8% (104) 38.8% (71)	62% (75) 31.4% (38)	46.8% (29) 53.2% (33)	0.06 **0.006**
NTproBNP (pg/mL) *	2756 ± 5319	2531 ± 4532	4158 ± 6502	**0.02**
GFR (mL/min)	46 ± 20	48 ± 21	42 ± 19	0.1
High-dose diuretics	44.8% (82)	38.8% (47)	56.5% (35)	**0.04**
No RAS inhibitor therapy	20.8% (38)	13.2% (16)	35.5% (22)	**<0.001**
**Periprocedural data**				
Procedure duration (min)	96 ± 38	90 ± 34	107 ± 42	**0.009**
General anesthesia	4.4% (8)	0% (0)	12.9% (8)	**<0.001**
Peri-interventional MR reduction (carpentier grade)	Δ2.1 ± 0.5	Δ2.1 ± 0.5	Δ2.0 ± 0.6	0.5
Length of postinterventional hospital stay (d)	7 (3)	6 (3)	8 (8)	**<0.001**
MACCE *Bleeding ≥ MVARC class II* *Cardiac death*	12.6% (23) *8.7% (16)* *2.7% (5)*	0% (0) *0% (0)* *0% (0)*	37.1% (23) *25.8% (16)* *9.3% (5)*	**<0.001** ***<0.001*** ***0.004***
**Echocardiographic characteristics**				
TR grade III	22.4% (41)	18.2% (22)	30.6% (19)	0.06
LVEF (%)	44 ± 12	45 ± 12	42 ± 13	0.07
Functional MR etiology	67.2% (123)	66.1% (80)	69.4% (43)	0.5
LA diameter (mm)	47 ± 10	46 ± 10	48 ± 10	0.4
LVEDD (mm)	56 ± 8	55 ± 8	57 ± 9	0.07
TAPSE (mm)	18 ± 4	18 ± 4	17 ± 4	0.2
TAPSE/PASP ratio	0.45 ± 0.18	0.46 ± 0.15	0.43 ± 0.23	0.5
Non-invasive PASP (mmHg)	43 ± 13	42 ± 12	45 ± 14	0.2
Invasive PASP (mmHg)	60 ± 18	55 ± 16	72 ± 18	**<0.001**

* Data presented as median ± IQR. Bold passages indicate subheadings and statistically significant *p*-values. Variables in italics represent subgroup variables. BMI—body mass index. CAB-OP—coronary artery bypass-OP. CAD—coronary artery disease. COPD—chronic obstructive pulmonary disease. CRT—cardiac resynchronization therapy. GFR—glomerular filtration rate. ICD—implantable cardioverter-defibrillator. LA—left atrium. LVEF—left ventricular ejection fraction. LVEDD—left ventricular end diastolic diameter. MACCE—major adverse cardiovascular and cerebrovascular events. MR—mitral valve regurgitation. M-TEER—transcatheter edge-to-edge mitral valve repair. MVARC—Mitral Valve Academic Research Consortium. NYHA—New York Heart Association. PAD—peripheral arterial disease. PASP—systolic pulmonary artery pressure. PCI—percutaneous coronary intervention. RAS—renin angiotensin system. TAPSE—tricuspid annular pulse systolic excursion. TR—tricuspid valve regurgitation.

**Table 2:** Independent factors that correlate with a required ICU treatment after successful M-TEER in multivariable logistic regression analysis.

Variable	Odds Ratio	95% Confidence Interval	*p*-Value
EuroSCORE II >10%	2.6	1.3–5.4	**0.006**
MitraScore >3	2.5	1.2–5.2	**0.02**
Hospital stay before M-TEER >5 days	3.2	1.6–6.4	**<0.001**

Bold values indicate statistically-significant *p*-values. ICU—intensive care unit. M-TEER—transcatheter edge-to-edge mitral valve repair.

**Table 3:** Independent factors that correlated with long-term mortality after multivariable Cox regression analysis.

Variable	Hazard Ratio	95% Confidence Interval	*p*-Value
ICD	2.2	1.4–3.7	**0.002**
High-grade TR	4.2	2.1–8.2	**<0.001**
TAPSE/PASP ratio > 0.5	0.3	0.1–0.7	**0.004**

Bold values indicate statistically-significant *p*-values. ICD—implantable cardioverter-defibrillator. PASP—pulmonary artery systolic pressure. TAPSE—tricuspid annular pulse systolic excursion. TR—tricuspid valve regurgitation.

## Data Availability

The raw data of the study are available upon request.

## Supplementary Materials

The following supporting information can be downloaded at https://www.mdpi.com/article/10.3390/jcm14072167/s1: Table S1: Independent predictors of a required ICU treatment after successful M-TEER in univariable logistic regression analysis; Table S2: Independent predictors of long-term mortality after univariable Cox regression analysis.

## Abbreviations

CAB	Coronary artery bypass
CAD	Coronary artery disease
COPD	Chronic obstructive pulmonary disease
CRT	Cardiac resynchronization therapy
GFR	Glomerular filtration rate
Hb	Hemoglobin
ICU	Intensive care unit
ICD	Implantable cardioverter-defibrillator
LVEDD	Left ventricular end diastolic diameter
LVEF	Left ventricular ejection fraction
MACCE	Major adverse cardiovascular and cerebrovascular events
MR	Mitral valve regurgitation
M-TEER	Transcatheter edge-to-edge mitral valve repair
MVARC	Mitral Valve Academic Research Consortium
NYHA	New York Heart Association
PAD	Peripheral arterial disease
PCI	Percutaneous coronary intervention
PCWP	Pulmonary capillary wedge pressure
RAS	Renin angiotensin system
PASP	Pulmonary artery systolic pressure
TAPSE	Tricuspid annular plane systolic excursion
TOE	Transesophageal echocardiography
TR	Tricuspid valve regurgitation
