# Supplementary material for: Predicting the Need for Intensive Care Unit Treatment After Successful Transcatheter Edge-to-Edge Mitral Valve Repair
Source: J Clin Med. 2025 Mar 22;14(7):2167. doi: 10.3390/jcm14072167 (PMC11989839; doi:10.3390/jcm14072167)
Supplement: Supplementary file 1 [file jcm-14-02167-s001.zip › jcm-3493853-supplementary.pdf]

Supplementary Table 1: Independent predictors of a required ICU treatment after successful M-TEER in univariable logistic regression analysis

Variable	Odds Ratio	95%-Confidence interval	p-value
euroSCORE II >10%	3.7	2.0-7.2	**<0.001**
STS-Risk-Score >10%	3.2	1.6-6.2	**<0.001**
NYHA class IV	2.5	1.3-4.7	**0.005**
MitraScore >3	3.6	1.9-7.4	**<0.001**
Persisting high-grade MR after M-TEER	4.3	1.7-12.1	**0.003**
Hb value <10g/dL	3.1	1.5-6.5	**<0.001**
GFR <45mL/Min	1.9	1.03-5.0	**0.04**
PCWP >30mmHg	2.9	1.2-7.6	**0.02**
PVR >4 Wood units	3.6	1.3-10.6	**0.02**
Hospital stay before M-TEER >5 days	3.9	2.0-7.4	**0.002**

GFR – glomerular filtration rate. Hb – hemoglobin. MR – mitral valve regurgitation. NYHA – New-York-Heart-Association. ICU – intensive care unit. PCWP – pulmonary capillary wedge pressure. PVR – pulmonary vascular resistance. M-TEER – transcatheter edge-to-edge mitral valve repair

Supplementary Table 2: Independent predictors of long-term mortality after univariable Cox regression analysis

Variable	Hazard Radio	95%-Confidence Interval	p-value
COPD	1.7	1.01-2.8	**0.04**
ICD	1.5	1.07-2.2	**0.02**
PAD	2.0	1.1-3.7	**0.02**
MitraScore >3	2.1	1.2-3-6	**0.006**
High grade TR	3.9	2.3-6.5	**<0.001**
TAPSE/PASP-ratio >0.5	0.4	0.2-0.9	**0.02**
Indication for ICU treatment after M-TEER	2.3	1.4-3.7	**0.001**

COPD – chronic obstructive pulmonary disease. ICD – implantable cardioverter defibrillator. ICU – intensive care unit. M-TEER – transcatheter edge-to-edge mitral valve repair. PAD – peripheral arterial disease. PASP – pulmonary artery systolic pressure. TAPSE – tricuspid annular pulse systolic excursion. TR – tricuspid valve regurgitation
